# A Machine Learning Pipeline for Prognostic Modeling of Alzheimer’s Disease Using Multimodal Data

**DOI:** 10.3390/s26051523

**Published:** 2026-02-28

**Authors:** Luisa De Palma, Vito Ivano D’Alessandro, Filippo Attivissimo, Anna Maria Lucia Lanzolla, Emilio Merlo Pich, Attilio Di Nisio, The Alzheimer’s Disease Neuroimaging Initiative, The Alzheimer’s Disease Metabolomics Consortium

**Affiliations:** 1 Department of Electrical and Information Engineering, Polytechnic University of Bari, Via E. Orabona 4, 70125 Bari, Italy; luisa.depalma@poliba.it (L.D.P.); v.dalessandro4@phd.poliba.it (V.I.D.); filippo.attivissimo@poliba.it (F.A.); attilio.dinisio@poliba.it (A.D.N.); 2 International Foundation of Artificial Intelligence and Big Data for Human Development, Via Galliera 32, 40121 Bologna, Italy; emilio.merlopich@ifabfoundation.org

**Keywords:** Alzheimer’s disease, XGBoost, prognostic modeling, features selection, multimodal biomarkers

## Abstract

Accurate prediction of progression to Alzheimer’s disease (AD) is crucial for early intervention and personalized patient management. In this study, we developed a robust, data-driven survival analysis pipeline to model time-to-progression from cognitively normal (CN) and mild cognitive impairment (MCI) at baseline to AD, integrating cognitive, clinical, MRI and PET neuroimaging biomarkers, and biospecimen features from the Alzheimer’s Disease Neuroimaging Initiative (ADNI) dataset. The ADNI cohort can be regarded as a multi-center platform for multimodal data integration that jointly captures cognitive performance, MRI/PET imaging-sensor biomarkers, and biofluid biosensing assays within a unified prognostic framework. Accordingly, our pipeline is designed to be robust to cross-site and cross-instrument variability through harmonized preprocessing and quality-check aware integration of heterogeneous multimodal data. Indeed, we employed eXtreme Gradient Boosting (XGBoost) for predicting survival data, which allows for the native handling of missing values that are frequently observed in real-world clinical datasets. Our results confirm that strong predictive performance can be achieved using a minimal set of features, obtaining a concordance index (C-index) of 0.92 using 13 features and 0.90 using only 4 features. These findings underscore the importance of multi-domain feature integration, transparent feature selection, and the inclusion of underexplored biomarkers such as lipid metabolites for prognostic modeling.

## 1. Introduction

Alzheimer’s disease (AD) represents one of the most pressing challenges in modern healthcare, affecting millions of individuals worldwide and imposing substantial burdens on patients, caregivers, and societies. Although symptomatic treatments exist and recent disease-modifying therapies have shown promise in slowing early cognitive decline, such as monoclonal antibodies targeting amyloid-β (e.g., lecanemab and donanemab), as well as non-pharmacological strategies including cognitive stimulation and lifestyle-based interventions, no curative therapy is currently available [1]. In this context, predicting the transition from Mild Cognitive Impairment (MCI) to AD has become one of the major topics of this field, since it can support individualized prognosis, facilitate enrollment in clinical trials, and enable the timely implementation of therapeutic and care strategies aimed at delaying progression or mitigating symptoms.

The evolution from MCI to AD can be viewed as a continuum, in which a window of opportunity exists for intervention before irreversible neurodegeneration and cognitive decline become advanced. Multimodal models combining biomarkers, brain imaging, and cognitive measures have shown strong predictive ability for this transition [2,3]. Recent systematic reviews have identified age, apolipoprotein E genotype 4 (APOE4), hippocampal atrophy, Mini-Mental State Examination (MMSE) score and Alzheimer’s Disease Assessment Scale cognitive (ADAS-Cog) score as consistent predictors of progression [4].

Complementing these findings, other authors have proven that integrating advanced machine learning (ML) models, especially in longitudinal and multi-variable contexts, can substantially enhance individualized prognosis and support precision medicine strategies in dementia [5,6] and also in other fields, such as in pancreatic diseases [7], lung adenocarcinoma [8] and liver cancer [9]. A substantial body of research has sought to identify predictive factors and develop models for conversion from MCI to AD. Early work focused on neuropsychological tests [10] and on neuroimaging highlighting that volumetric reductions in hippocampus, entorhinal cortex, parahippocampal regions and other medial temporal structures areas early markers of conversion [11]. More recently, systematic reviews have evaluated the growing number of ML and multimodal studies underlying that the studies achieving highest performance tend to integrate multimodal data (imaging, clinical, biofluid) and sophisticated classifiers [12,13]. Structural imaging studies reinforce the value of region-specific biomarkers [14,15,16]. Recent advances in AD diagnostic workflows have also explored efficient deep learning frameworks that enhance imaging-based classification performance. For example, Huang et al. [17] proposed a token-efficient Vision Transformer architecture that partitions structural MRI scans into patch tokens and employs a Patch Residual Block to improve focus on diagnostically relevant regions, demonstrating superior diagnostic accuracy and generalization across multiple large datasets.

Beyond performance metrics, the intrinsic heterogeneity of AD further underscores the need for improved predictive models. Reported annual conversion rates from MCI to AD typically range from 10% to 15%, but they can vary substantially across studies due to differences in cohort composition, diagnostic criteria, and follow-up duration. This variability highlights the importance of developing models capable of generalizing across diverse patient populations.

Despite the advances, several limitations remain pervasive in the field. Many studies rely on the ADNI cohort, which may not fully represent the heterogeneity of clinical populations. External validation in diverse, real-world patient groups is still relatively rare. The variety of preprocessing pipelines, follow-up durations, and conversion definitions impede direct comparison across different studies. Systematic reviews emphasize the need for improved calibration of predictive models, broader external validation, and transparent reporting of feature importance and interpretability [4]. Furthermore, while many models demonstrate high discrimination, their translation into clinical decision-making frameworks remains limited, partly due to complexity, black-box nature, and issues of generalizability.

In addition, many previous studies omit a dedicated feature selection phase, fail to report the exact number and type of retained predictors, or do not assess feature stability across validation folds. Furthermore, multimodal datasets are frequently handled through imputation procedures that may introduce bias and limit reproducibility. At the same time, the multifactorial nature of AD progression requires the comprehensive integration of heterogeneous information.

In recent years, XGBoost has become one of the most popular and powerful ML algorithm in the biomedical signal and biomarker research literature due to its ability to handle structured tabular feature sets, capture non-linear relationships and interactions, provide built-in regularization and ranking of feature importance, and accommodate heterogeneous data types. Its flexibility and predictive performance have been demonstrated across a range of applications, from Photoplethysmography (PPG)-based blood pressure estimation [18,19] to survival prediction in oncology [20,21] and neurological diseases [22]. In survival analysis, adaptations of XGBoost for time-to-event data have shown improved performance over conventional Cox models and other ML approaches, enabling robust modeling of censored data and complex biomarker interactions. These findings support the use of XGBoost in our study, where we embed a Cox proportional-hazards framework within XGBoost to predict AD progression, leveraging the model’s capacity to integrate cognitive, radiomic, and clinical features while capturing non-linear relationships that classical linear models may overlook.

The current work seeks to address several of the reported gaps by implementing a robust survival-analysis framework using XGBoost with feature selection and hyperparameter tuning, applied to a well-characterized Alzheimer’s dataset. Unlike many previous studies that focus exclusively on the MCI-to-AD transition, we explicitly consider all conversion pathways leading to AD, including cognitively normal (CN)-to-MCI-to-AD, direct CN-to-AD, and MCI-to-AD trajectories. First, we incorporate all available feature domains, including clinical, cognitive, genetic, and imaging data, within a unified framework, aiming to capture the complex and multidimensional mechanisms underlying disease evolution while maintaining methodological transparency and robustness.

Secondly, we evaluate whether competitive prognostic performance, measured in terms of C-index, can be achieved using a reduced and stable feature set derived from multimodal data without imputation. Additionally, we investigate the independent and complementary contributions of cognitive and radiomic domains by analyzing them separately, thereby providing insight into their respective prognostic roles.

Particular attention was devoted to ensuring the robustness and reproducibility of the proposed framework. The dataset was divided into training and test sets maintaining balanced event representation. The training set was further partitioned into stratified cross-validation folds, and feature stability was explicitly assessed by retaining only predictors consistently selected across folds, reducing variability due to data partitioning and mitigating overfitting driven by fold-specific selection. Strict separation between training and test data was enforced throughout the entire pipeline to prevent data leakage. Finally, no imputation procedures were applied, preserving the intrinsic structure of the multimodal dataset and avoiding potential biases introduced by artificial data completion.

In doing so, we aim to contribute a methodologically transparent and clinically relevant prognostic tool for AD conversion, complementing existing literature and advancing toward clinical implementation.

The structure of the paper is as follows. In Section 2, the dataset utilized in this study is presented. In Section 3, the development of the prognostic model is discussed, along with the hyperparameter tuning, the examination of the features selection step and the model’s testing. In Section 4, the results are shown. Finally, the findings are discussed in Section 5, and conclusions are drawn.

## 2. Dataset

Data used in the preparation of this article were obtained from the Alzheimer’s Disease Neuroimaging Initiative (ADNI) database (adni.loni.usc.edu). This dataset was launched in 2003 as a public–private partnership, led by Principal Investigator Michael W. Weiner, MD. The primary goal of ADNI has been to test whether serial MRI, PET, other biological markers, and clinical and neuropsychological assessment can be combined to measure the progression of MCI and early AD.

In the present study, all data were retrieved from the ADNI database and organized into five major feature domains, each representing a complementary aspect of the disease phenotype.

- *Cognitive domain* includes neuropsychological and clinical test scores that capture cognitive and functional performance. Key features comprise the MMSE, ADAS-Cog, Clinical Dementia Rating—Sum of Boxes (CDR-SB), Functional Activities Questionnaire (FAQ), and other standardized assessments of memory, language, and executive function.
- *Biochemical and biosensing domain* contains quantitative data from blood and cerebrospinal fluid (CSF) analyses, including concentrations of β-amyloid (Aβ), total tau, phosphorylated tau (p-tau), and other plasma and metabolomic markers such as lipids and phospholipids. These measures provide biochemical insights into the molecular mechanisms of AD pathology.
- *Genetic domain* incorporates genotyping and genomic information such as the APOE genotype, along with other single nucleotide polymorphisms (SNPs) known to influence AD risk or disease progression. These features capture inherited susceptibility and interindividual variability in biological pathways related to amyloid and lipid metabolism.
- *Imaging domain* includes quantitative MRI and PET-derived measures encompassing structural, functional, and metabolic imaging biomarkers. Examples include hippocampal and cortical volumes, cortical thickness, ventricular enlargement, amyloid and derived indices such as the hippocampal convergence index (HCI). These imaging features reflect the neurodegenerative and metabolic changes associated with AD progression.
- *Subject characteristics domain* contains demographic and clinical background variables such as age, sex, education, and race.

By integrating these five domains, the dataset captures the multifactorial nature of AD and allows a comprehensive modeling of disease progression. This multimodal structure not only enhances predictive performance but also promotes interpretability by enabling domain-specific analyses of feature relevance.

All data were stored in tabular format and retrieved as distinct CSV files, each corresponding to a specific domain and ADNI enrollment phase. These files were subsequently standardized, processed, and merged to construct the unified dataset used for analysis.

### 2.1. Processing and Merge

The quality and comprehensiveness of a dataset is crucial not only in AD research but across all fields [23,24], as robust, well-characterized data enable reliable modeling, accurate predictions, and meaningful biological insights.

Since its inception, ADNI has evolved through multiple phases, ADNI1, ADNIGO, ADNI2, ADNI3, and ADNI4, each introducing specific methodological innovations and technological advancements:

1. ADNI1 (2004–2010) established the foundational framework for data collection and standardization. Approximately 800 participants were enrolled, including CN individuals, subjects with MCI, and patients diagnosed with AD. This phase introduced amyloid PET imaging, structural MRI for brain morphology assessment, and CSF biomarker analysis for β-amyloid and tau quantification. Participants were followed for an average of 2–4 years with periodic clinical and neuropsychological evaluations.
2. ADNIGO (2009–2013) extended ADNI1 by recruiting approximately 200 additional older participants at elevated risk for AD, aiming to refine the identification of early biomarkers and enhance the sensitivity of preclinical detection.
3. ADNI2 (2011–2017) further expanded the cohort with around 1000 new subjects and incorporated advanced neuroimaging modalities, such as tau PET, to capture longitudinal changes in neurofibrillary pathology.
4. ADNI3 (2017–2022) is the most recent large-scale continuation of the study, emphasizing high-resolution imaging technologies, enhanced biomarker precision, and the integration of genetic profiling to identify individuals at risk of developing AD even before the onset of clinical symptoms. Over 1500 participants have been enrolled across the full clinical spectrum, from CN to MCI and AD.
5. ADNI4 (2022–present) builds on the extensive legacy of the ADNI project, aiming to increase the representativeness and generalizability of ADNI data to the broader North American population. The phase began participant enrollment in 2023 and involves over 60 research centers across the United States and Canada. ADNI4 will recruit up to 1500 participants, including elderly controls, individuals with MCI, and patients with AD or other dementias. This phase places particular emphasis on ensuring demographic diversity and inclusivity, while continuing to refine and validate clinical trial measures and biomarkers for Alzheimer’s research.

Across these phases, data acquisition protocols, feature definitions, and variable naming conventions have evolved substantially. Key identifiers such as VISCODE2 (visit code) and several feature labels were redefined or extended to accommodate new data formats and technologies. As a result, direct merging of data across ADNI phases is not straightforward and requires a rigorous standardization and cleaning process. Thus, in our work, for each domain, the corresponding CSV files containing the features were carefully preprocessed and standardized prior to integration. Initially, all non-informative variables were removed, including columns unrelated to disease status or progression, such as instrument models, PET field strength, acquisition protocol identifiers, or administrative metadata.

Subsequently, missing or non-quantifiable values were standardized across all files using a unified mapping. In the ADNI dataset, such values are represented through heterogeneous encodings, including numeric flags (e.g., −4), symbolic placeholders (e.g., “-”), and inequality expressions (e.g., “<1”, “<3”, “<9”, “<20”), as well as other non-numeric entries such as “*QNS” (“Quantity Not Sufficient”), which means that the biological sample was inadequate for accurate testing, typically due to extraction errors, sample depletion, or insufficient collection.

$$NaN_{ALIAS}=[-4,‘-’,‘<1’,‘<3’,‘<4’,‘<9’,‘<20’,‘\#VALUE!’,‘<1.0’,‘*QNS’]$$

Since these formats cannot be used directly in ML algorithms, all corresponding entries were uniformly converted to NaN .

Feature name aliases were then harmonized to maintain consistent nomenclature across domains, ensuring that equivalent variables differing only in suffix or capitalization shared a single standardized name. Additionally, columns and rows consisting entirely of NaN were removed.

Records missing either the RID (unique participant identifier) or VISCODE2 were excluded, as these fields are essential to merge all the CSV files into a unified dataset.

Particular attention was given to the standardization of the VISCODE2 variable, which originally contained mixed alphanumeric labels describing visit timing, such as *sc* (screening), *scmri* (screening MRI), *bl* (baseline), *m12* (month 12), *nv* (no visit defined), and *f* (failed screen). In the ADNI3 and ADNI4 phases, additional codes such as *y1*, *y2*, and others were introduced to indicate annual follow-ups. To harmonize this field, *sc* and *scmri* were converted to *bl*, while rows labeled as *nv* or *f* were removed. Afterwards, visit codes were numerically encoded to represent the number of months since baseline (e.g., *bl* → 0, *m12* and *y1* → 12, *m24* and *y2* → 24, and so on).

For the cognitive assessment data, we standardized the column names for the ADAS-Cog 11 and ADAS-Cog 13 scores across all participants to ensure consistency. More-in-depth, two columns contained the same information but were labeled differently because they originated from different ADNI phases. These were therefore consolidated into a single unified variable encompassing all phases. The same harmonization procedure was applied to the individual task, which also appeared under different names across phases despite representing identical assessments.

In addition, we derived several features from the Rey Auditory Verbal Learning Test (RAVLT), including RAVLT immediate recall, RAVLT learning, RAVLT forgetting and RAVLT percent forgetting scores. These computed features capture different aspects of memory performance and were integrated into the dataset for downstream survival analysis.

Categorical variables, including demographic and clinical information such as sex and race were subsequently transformed into numerical labels using a label-encoding procedure.

After the completion of all preprocessing operations, the cleaned domains were aligned by RID and VISCODE2 and merged into a single unified dataset. This dataset contains 2265 participants who all had at least one valid baseline visit. At baseline, 852 individuals were classified as CN, 1034 were classified as MCI, and 379 were classified as AD. The merged dataset contains six static covariates (sex, education, ethnicity, race, age and marital status), and 31,997 are longitudinal covariates collected over time. Indeed, excluding non-feature identifiers such as the diagnosis field, the visit codes, the participant identifier and the study phase, the dataset comprised 32,003 usable features for survival modeling or analysis of disease progression.

The temporal structure of the dataset was heterogeneous across participants. The number of visits per individual varied substantially and produced a follow-up window spanning from zero months at minimum to 228 months at maximum, with an average follow-up duration of approximately 57 months. Baseline demographic characteristics indicate that participants have a mean age of 73.1 years (SD = 7.3), with ages ranging from 50.6 to 91.6 years. The sex distribution is relatively balanced, with 52.9% of participants identified as male and 47.1% as female. Furthermore, the education level is generally high, with an average of 16.1 years and a range from 4 to 20 years.

### 2.2. Dataset Stratification by Clinical Diagnosis and Event

This work focuses on the importance of distinguishing patients who convert to AD from those who remain stable and do not progress to dementia. Identifying converters at an early stage is crucial for understanding the mechanisms underlying disease progression and for improving prognostic accuracy. For this reason, only baseline data were used to ensure a more realistic and applicable approach in real-world scenarios. Patients with a baseline diagnosis of CN or MCI were included in the analysis. The occurrence of an event was defined by a complete conversion to AD as detailed in Table 1. In cases where no conversion took place, the event was considered absent, and the observation period was defined as the time until the patient’s most recent follow-up visit. This approach allows for the incorporation of censored data while maintaining consistency with standard survival analysis frameworks.

Based on the event definition described above, the transition rules were applied to the entire dataset to construct the survival cohort. The initial dataset consisted of 2265 participants; among them, 1886 individuals had a baseline diagnosis of either CN or MCI and were therefore retained for the progression analysis. Within this cohort, 434 individuals experienced a confirmed conversion to AD and were classified as events, whereas the remaining 1452 participants did not convert to AD during follow-up and were treated as right-censored observations in accordance with standard survival analysis methodology. Among converters, the mean time to AD was 38.20 months, with a standard deviation of 32.85 months.

## 3. Models and Methods

In this study, we developed a data-driven survival analysis pipeline to investigate progression patterns in subjects from the ADNI dataset. The goal was to model time-to-progression toward AD using a gradient boosting framework for survival data (XGBoost—Cox model), while ensuring balanced event representation, robust feature selection, and hyperparameter optimization. XGBoost was specifically chosen for its ability to naturally handle missing values (NaNs), allowing the analysis to be conducted without any imputation methods, which preserves the integrity of the original data and avoids potential biases introduced by filling missing entries. In this study, we employed the XGBoost-Cox framework to model survival outcomes; this approach combines the Cox proportional hazards model with gradient-boosted trees, enabling the algorithm to automatically capture complex, non-linear relationships among covariates and interactions that are not easily represented in a traditional Cox model. Unlike standard Cox regression, XGBoost-Cox does not strictly rely on the proportional hazards assumption for accurate prediction, as its tree-based structure can flexibly accommodate time-varying and non-linear effects. This allows the model to improve predictive performance and capture patterns that may be missed by a purely linear approach.

To ensure reproducibility, robustness, and prevention of data leakage, the entire ML pipeline was structured into sequential and clearly separated stages: data partitioning, hyperparameter optimization, cross-validated feature selection, model stability assessment, and independent testing.

All preprocessing steps were implemented in Python (v3.11), using pandas, scikit-learn, and XGBoost libraries.

### 3.1. Data Partitioning Strategy

The dataset was first cleaned by removing constant or redundant variables and harmonizing column names, as previously discussed. Observations missing either the event indicator or survival time (time_months, event) were excluded to ensure valid survival targets. To prevent outcome imbalance, we applied a stratified shuffle split maintaining the same proportion of events in both training (90%) and test (10%) subsets. This stratification ensured comparable baseline risk distributions across data partitions, with a final event rate of approximately *p*(*event*)* ≈ constant* across subsets as shown in Table 2. The test set was strictly held out and was not involved in any stage of hyperparameter tuning, feature selection, or model development.

Then, we applied stratified 5-fold cross-validation on the training set to train the survival model, as shown in Table 3.

### 3.2. Hyperparameter Optimization

To enhance the model’s predictive performance hyperparameters were optimized using the Optuna framework with stratified cross-validation to preserve the event rate across folds ensuring that the percentage of events in each fold remained within ±1% of the overall event rate (controlled via a tolerance parameter) as shown in Table 3. Performance was evaluated using the C-index, which measures the agreement between predicted risk scores and observed event times. A pair of observations is considered concordant if the patient with the higher predicted risk experiences the event before the patient with the lower predicted risk. The metric is implemented in the lifelines Python package.

The best parameter set was selected based on the highest mean C-index on the validation folds over 30 trials. Each Optuna trial optimized the following key parameters of XGBoost as shown in Table 4.

The model was trained using the survival:cox objective, which minimizes the negative partial log-likelihood of the Cox proportional hazards model. By optimizing this function, XGBoost effectively relates covariates to the hazard function while handling censored survival data and capturing potential nonlinear effects. Model performance was assessed separately on a validation set using the C-index, which evaluates the model’s ability to correctly rank survival times and provides a measure of discriminative power.

### 3.3. Cross-Validated Feature Selection and Stability Assessment for Model Robustness and Generalization

To assess model stability and generalization, we applied the same 5-fold stratified cross-validation procedure using the survival:cox objective and the optimal hyperparameters identified previously. Within each fold, an initial XGBoost model was trained on all available features from different domains to compute feature importance scores. Features were then ranked by importance, and new models were retrained using progressively smaller subsets of the top-k ranked features, where

k∈{20,000;15,000;10,000;5000;1000;500;100;50;20;10;5}

This hierarchical selection enabled quantifying the trade-off between model complexity and predictive performance.

For each configuration, the mean C-index and standard deviation across folds were recorded, and the intersection of top-ranked features across folds was identified as the stable predictive subset, to ensure feature robustness and minimize variability due to sampling effects. This intersection-based strategy ensured that selected predictors were not fold-specific artifacts but represented reproducible prognostic signals.

### 3.4. Final Model Development and Independent Testing

Using optimal hyperparameters and stable feature subset, a final XGBoost survival model was trained on the entire training dataset. The model was then evaluated on the held-out test set, where the C-index served as the primary model performance metric. Because the test set was not used during hyperparameter tuning or feature selection, this evaluation provides an unbiased estimate of generalization performance.

**Figure 1:**
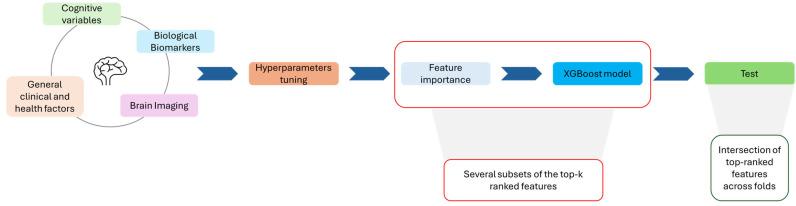
Pipeline of the ML framework for survival prediction in AD.

The pipeline shown in Figure 1 provides a comprehensive and reproducible ML framework for survival prediction in AD, integrating robust statistical validation, automated hyperparameter tuning, and interpretable feature selection. By embedding the Cox proportional hazards formulation within XGBoost, the model effectively captures nonlinear biomarker interactions that are often missed by classical linear survival models. Moreover, balancing event proportions across folds ensures reliable generalization and mitigates bias due to unequal censoring distributions.

### 3.5. Multidomain and Domain-Specific Analyses

The same feature selection and modeling pipeline described above was consistently applied across all analyses in this study. Using this approach, we trained models on the full set of multimodal features to evaluate potential synergistic effects among cognitive, radiomic, clinical, genetic, and biospecimen domains in predicting disease progression. To further investigate domain-specific contributions, the identical pipeline was applied to individual feature subsets, including cognitive-only and radiomic-only features. In these analyses, models were trained exclusively on each domain to assess the discriminative power of neuropsychological measures and the prognostic information provided by neuroimaging-derived variables, respectively. By applying a unified procedure to both integrated and domain-specific analyses, we ensured methodological consistency, comparability, and robustness, enabling a comprehensive evaluation of both synergistic and independent contributions of the different feature domains.

## 4. Results

As previously described, a fully integrated model using all available features was trained without feature selection to assess the cumulative contribution of all variables to model performance as shown in Table 5.

However, considering the model complexity and the challenges associated with obtaining all features, we implemented the pipeline described above with the goal of reducing the number of features required to train a high-performing model, while also simplifying the feature acquisition process.

For this reason, all possible configurations of top-ranked features were evaluated to identify those with a reduced yet stable feature intersection that still achieved a competitive C-index on the test set. When varying the number of top features considered, the best model performance was achieved with a relatively small subset of features. Specifically, when analyzing the 500 most important features identified in each of the five cross-validation folds, which results are shown in Table 6, only 27 features were found to be common across all folds. Despite their limited number, these 27 shared features proved to be highly representative and informative, yielding improved C-index compared to the model trained using the 500 most important features, as can be seen in Table 7. This finding suggests that these features effectively capture the most relevant information for the problem under study, providing strong predictive power while significantly reducing model complexity.

The results reported in the table represent the C-index values obtained across the five folds using 500 features. When using the 27 features identified as the intersection across folds, the corresponding C-index values for the five folds are shown in Table 7.

**Figure 2:**
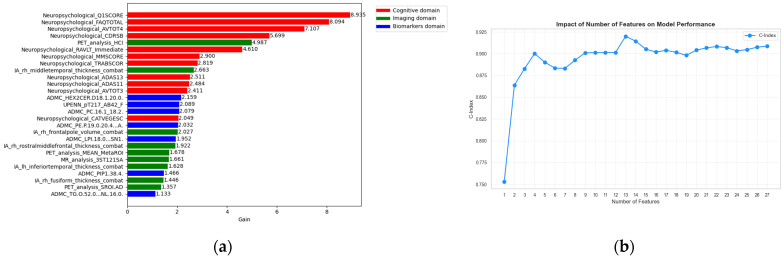
(**a**) Feature importance ranking from the XGBoost survival model across cognitive, imaging, and biospecimen domains. The predominance of cognitive variables reflects their central role in clinical assessment of disease severity and progression risk, while selected imaging and biospecimen features provide complementary prognostic information.; (**b**) Effect of the number of features on model performance (C-index), showing that reliable risk stratification can be achieved using a limited set of baseline features, supporting parsimonious and interpretable prognostic modeling.

In Figure 2a the feature importance plot is shown in order to identify the most significant features among the 27 previously identified features. An interesting aspect regarding the stability of these features is the analysis performed by sequentially adding features in each iteration to assess their impact on the C-index as shown in Figure 2b. We observed that using only the top 4 features the model already achieves a C-index of 0.90, while using the top 13 features yields the best performance of 0.92. Moreover, even with as few as 4 features the model attains an high C-index, which is comparable or greater than the values commonly reported in the literature [25,26,27,28,29,30].

**Figure 3:**
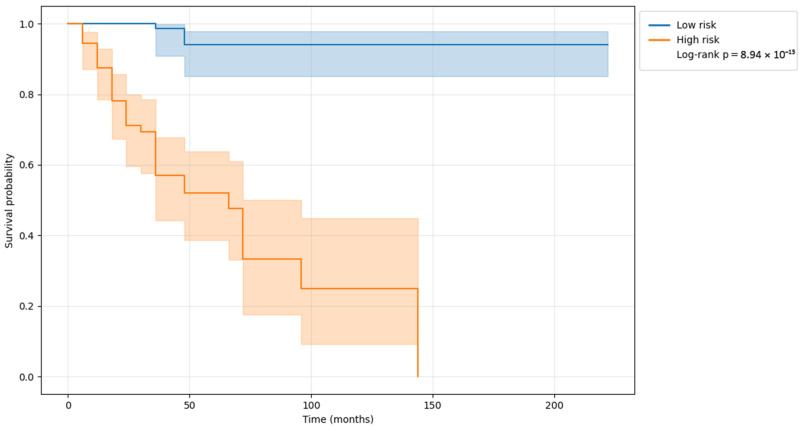
Kaplan–Meier survival curves stratified by predicted risk groups. Shaded areas indicate the 95% confidence intervals. The clear separation between low- and high-risk groups indicates that the model can meaningfully stratify individuals according to progression risk over time, supporting its use for prognostic risk stratification.

Beyond discrimination performance, the clinical interpretability of the model predictions was further investigated by analyzing survival outcomes associated with the estimated risk scores. Figure 3 shows Kaplan–Meier survival curves stratified according to the predicted risk, using the median risk score derived from the training dataset as the cutoff, highlighting a clear separation between risk groups and confirming the model’s ability to meaningfully differentiate patients with distinct survival profiles.

**Figure 4:**
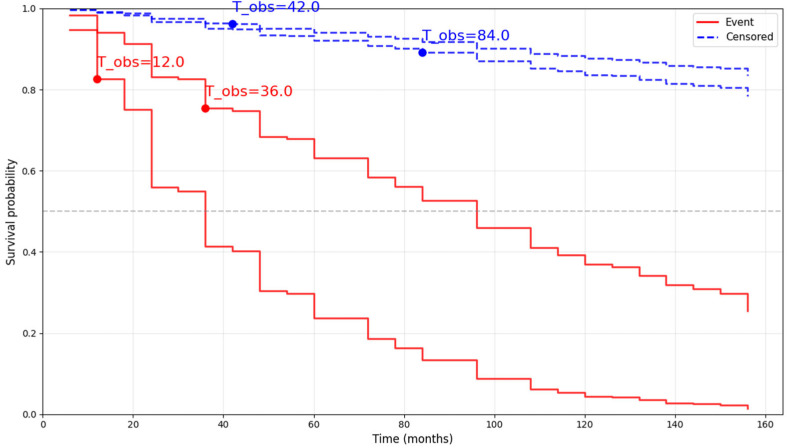
Individual survival curves for four representative patients from the test set, including two subjects with observed progression events (red lines) and two censored subjects (blue dashed lines), estimated using baseline covariates only. The curves illustrate patient-specific differences in progression risk over time, supporting individualized risk profiling within a prognostic framework.

To further illustrate the personalized nature of the proposed approach, individual survival curves for four representative patients from the test set are reported in Figure 4. Two of these patients experienced the event, while the remaining two were censored, allowing a qualitative comparison between predicted survival trajectories and observed outcomes. These visualizations provide an intuitive link between the model’s risk estimates and clinically interpretable time-to-event predictions, thereby enhancing the translational relevance of the proposed pipeline.

### 4.1. Cognitive Features Domain Analysis

An analogous analysis was performed focusing exclusively on the cognitive feature domain, comprising 143 variables. Employing the hyperparameters optimized during the tuning phase considering all the features available, the best performance was achieved by selecting the top 50 cognitive features within each cross-validation fold. The intersection of the selected features across folds resulted in 15 common features, which produced strong discriminative performance on the test set, as summarized in Table 8.

**Figure 5:**
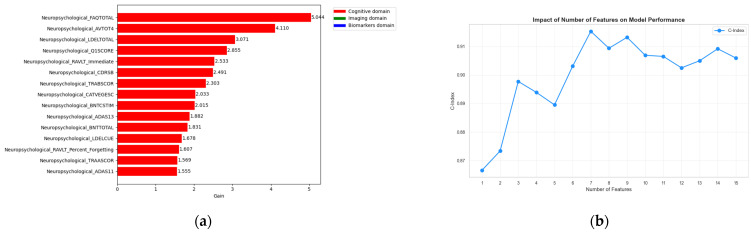
(**a**) Feature importance ranking from the XGBoost survival model considering cognitive covariates only; (**b**) Effect of the number of cognitive features on model performance (C-index), showing that reliable risk stratification can be achieved using a limited set of baseline cognitive measures.

In Figure 5a the feature importance plot is shown in order to identify the most significant features. An interesting observation regarding feature stability emerged from the analysis in which features were sequentially added across iterations to evaluate their incremental impact on the C-index. The results indicate that using only the top 7 features produced the best performance of 0.9153 as shown in Figure 5b.

### 4.2. Radiomic Features Domain Analysis

An analogous analysis was carried out on the radiomic feature domain, consisting of 13,196 variables. With the optimized hyperparameters from the tuning phase, the model achieved its best performance when the top 100 features were selected in each fold. The overlap of selected features across folds resulted in 9 recurrent features and the model performance on the test set are presented in Table 9.

**Figure 6:**
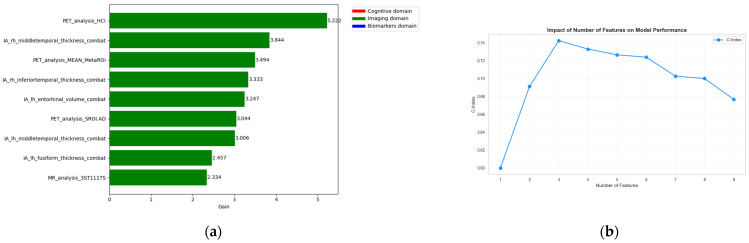
(**a**) Feature importance ranking from the XGBoost survival model considering imaging-derived covariates only; (**b**) Effect of the number of imaging features on model performance (C-index), illustrating that imaging-only models provide more limited risk stratification compared to cognitive-based models.

An insightful finding regarding feature stability arose from the analysis where features were progressively introduced at each iteration to assess their incremental contribution to the C-index as shown in Figure 6. The optimal performance was observed when the first three features were included, obtaining 0.7425.

As we can observe, radiomic features alone are less predictive than cognitive features. In fact, cognitive features achieve the best possible performance using only seven features, which is expected since, without domain separation, cognitive features are naturally selected as the most significant ones.

### 4.3. Radiomic and Cognitive Features Domain Analysis

**Figure 7:**
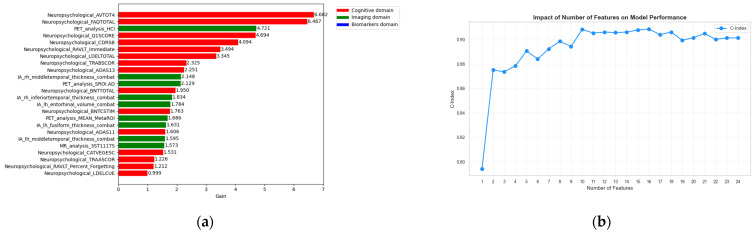
(**a**) Feature importance ranking from the XGBoost survival model integrating baseline features from cognitive and imaging domains; (**b**) Effect of the number of cognitive and imaging features on model performance (C-index).

Training a new XGBoost model on the 9 radiomics and 15 cognitive variables identified in the previous paragraphs, the obtained feature importance is shown in Figure 7a Moreover, it is worth noting that training on only the first 10 most important features, yield a C-index of 0.9082 as shown in Figure 7b.

However, when comparing this result with that obtained using cognitive features alone, it becomes evident that introducing two radiomic features among the seven most important leads to a reduced C-index.

## 5. Discussion

This work focuses exclusively on baseline data, which enables the development of a prognostic model that can be applied early in the disease course, before longitudinal follow-up is available. By relying on initial assessments, the model supports timely risk stratification and individualized monitoring, demonstrating that meaningful predictions can be achieved even with a single time point. This approach emphasizes the clinical utility of baseline measurements within a prognostic framework, showing that early data alone can provide robust insight into disease progression. The dataset obtained through the processing steps includes all available patients. We retained observations with missing values, thus maximizing data utilization and ensuring a comprehensive representation of the studied population.

A 5-fold cross-validation procedure was applied to the training set to systematically assess the impact of varying feature subsets on predictive performance. To enhance feature stability and reproducibility, only those variables consistently selected across all folds were retained to train the final model, which was subsequently evaluated on the independent test set. This strategy enabled a reliable assessment of model generalizability and yielded promising predictive results. Notably, the model achieved a C-index of 0.92 using only 13 features, and a C-index of 0.90 with a minimal subset of 4 features.

A key finding of this study concerns the different predictive capabilities of cognitive and radiomic domains. When the model was trained exclusively on radiomic features, the resulting C-index was considerably lower than that obtained using cognitive features alone. This suggests that radiomic descriptors, while rich and high-dimensional, offer limited prognostic power when used alone. On the other hand, cognitive scores directly reflect early functional changes that precede dementia and are therefore stronger predictors of imminent decline. Importantly, cognitive assessments are substantially easier, faster, and more cost-effective to obtain than radiomic biomarkers, which require MRI or PET imaging, segmentation workflows, and radiomic extraction pipelines.

These findings were further confirmed by analyzing the predictive performance of models trained on both cognitive and radiomic features: cognitive-related variables consistently emerged as the most influential predictors, reinforcing their central role in forecasting disease progression. However, these results should be interpreted considering the inherent clinical circularity associated with several of the high-ranking predictors identified in this study. Cognitive covariates such as CDR-SB, FAQ, ADAS-Cog, and RAVLT-derived measures are highly correlated, and in some cases directly involved in, the clinical criteria used to define diagnostic conversion to AD. As a result, their strong predictive contribution partly reflects their role within the diagnostic framework itself, rather than representing fully independent biological predictors of disease onset. Therefore, the high reported C-index reflects the model’s ability to consistently rank progression risk among individuals already undergoing clinical assessment, rather than identify preclinical disease in the absence of clinical information.

While radiomic features provide additional, objective disease-related signals that are independent of cognitive testing, potentially enhancing model robustness and generalizability by capturing structural patterns. From a practical perspective, the multi-domain framework allows flexible deployment: cognitive-only models can be employed for rapid and cost-effective screening, while the inclusion of selected radiomic features can support decision-making in settings where imaging data are already available. This modular design thus balances predictive performance, feature complexity, and clinical utility, and provides a clear rationale for integrating radiomics even when the incremental gain in discrimination appears modest.

A further limitation of this study concerns generalizability. All stages of model development, hyperparameter tuning, and evaluation were conducted exclusively within the ADNI cohort. Consequently, the reported performance metrics primarily reflect robustness within a standardized research environment and should not be interpreted as demonstrating assured applicability across heterogeneous real-world clinical settings. As reported in the literature, several of the features selected in our study have been recognized as significant predictors of progression to dementia, including CDR-SB [31], FAQ [32], and PET HCI [33], with similar relevance observed for some of the radiomic features [34]. Notably, among the lipid metabolites, our model specifically selected ADMC_HEX2CER.D18.1.20.0. representing a glycosphingolipid Hex2Cer(d18:1/20:0), which is a dihexosylceramide or lactosylceramide (a ceramide with two sugar units attached) measured in blood plasma samples. The connection between lipid dysregulation in the human brain and AD have been studied [35,36,37]. When combined with cognitive features only, inclusion of Hex2Cer(d18:1/20:0) slightly reduced the C-index from 0.9059 to 0.9037. However, the feature was consistently present in the intersected selected features in 7 out of 9 top ranked sets, indicating relatively robust inclusion, although its presence is somewhat sensitive to the feature-ranking threshold. Moreover, among the cognitive features, ADAS-Cog 13 and ADAS-Cog 11 have been consistently recognized as significant predictors [38,39], a finding that is also supported by our analysis. Also Rey Auditory Verbal Learning Test-Trial 4 (AVTOT4) and 3 (AVTOT3) but also the RAVLT immediate score have been recognized as significant features [28]. An interesting observation is that the features identified as most significant in our study substantially overlap with those reported in [40]. However, this similarity does not indicate a lack of novelty. Instead, it reinforces the robustness of our results, especially considering that our methodological framework introduces several improvements over the state of the art. Unlike [40] our analysis was conducted on the full set of available participants, retaining subjects with missing values thanks to the native handling of missingness offered by XGBoost. Additionally, our processing pipeline differs substantially in both feature preparation and model evaluation, demonstrating that the importance of these biomarkers is consistent across independent methodological approaches.

Moreover, while the authors of [40] include cognitive composite scores such as ADNI_MEM and ADNI_EF, as indicated in their Supplementary Materials, these variables are derived from longitudinal neuropsychological assessments and are specifically designed to capture cognitive change over time, rather than baseline status. In contrast, our work explicitly excludes all longitudinal composite scores and harmonized cognitive variables, including ADNI_MEM and related measures. This choice ensures that our analysis remains strictly a baseline-only framework, eliminating any form of temporal leakage and preserving both temporal validity and interpretability of the results.

By relying solely on baseline data, using a larger sample, and improving methodological rigor while still recovering a biologically coherent subset of the features identified in prior work, our results support the stability and relevance of these biomarkers within a clinically grounded prognostic setting. Importantly, the proposed multimodal fusion and ML framework also demonstrates clear clinical deployment potential. Its interpretable and stable predictions can guide clinicians in early identification of high-risk individuals, supporting timely intervention strategies and patient stratification for clinal trials. The framework’s flexibility in integrating cognitive, radiomic, clinical, genetic, and biospecimen data allows adaptation to diverse clinical datasets, while the ability to perform domain-specific analyses help clinicians understand the relative contribution of each type of measurement. Together these features suggest that the model could be a practical decision-support tool to inform individualized monitoring and treatment planning in real world clinical settings.

### Literature Comparison

Several studies in the literature have investigated predictive modeling in AD, particularly focusing on the conversion from MCI to AD or cognitive decline in preclinical stages. C-index is commonly used to evaluate the discriminative ability of these models.

For example, a study integrating multi-omics data, including miRNA and genomic features, reported a C-index of 0.702 on an independent test set for predicting MCI-to-AD conversion [5]. Using imaging data, another work applied deep survival analysis to MRI scans, achieving a C-index up to 0.835 when combining gray matter volume, age, and MMSE scores [25].

Large cohort studies, such as those based on the ADNI dataset, have demonstrated even higher predictive performance. One recent study comparing traditional Cox models and RSF reported a C-index of 0.878 (95% CI: 0.877–0.879) for RSF models predicting MCI-to-AD conversion using 14 features [28]. Similarly, a ML approach integrating demographic, cognitive, and comorbidity information achieved a C-index of 0.84 [29] to predict transition from CN to MCI.

Some studies have explored ensemble approaches and longitudinal biomarker data, reaching even higher discriminative ability. For instance, RSF have been used with a focus on model explainability, showing an accuracy of 0.890 compared to 0.819 for traditional Cox models in MCI-to-AD conversion, though a C-index was not explicitly reported [26]. Another study [30] developed ensemble survival models to predict the progression from CN to MCI or AD using longitudinal biomarkers from the ADNI cohort. By combining baseline and follow-up data on cognition, genetics, CSF biomarkers, and brain imaging, the ensemble approach achieved 0.608 using baseline data alone. Similarly, in [41] the authors compared multiple ML approaches for survival analysis in high-dimensional dementia data, reporting a high C-index of 0.93 for the ADNI cohort. However, their study was based on a relatively small feature set of 207 variables and achieved this performance without applying any feature selection method. In contrast, our work leverages XGBoost feature importance to select relevant predictors, achieving a comparable C-index of 0.90 while using only 4 features from a much larger initial feature set and 0.92 using 13 features.

Regarding the specific conversion analyzed in this paper, few studies have addressed this type of stratification. For instance, in [42] the authors developed a prognostic model to predict conversion to AD using functional connectivity derived from resting-state fMRI. CN and MCI participants from the ADNI dataset were analyzed, with functional connectomes mapped using manifold learning to generate cortical and subcortical gradients. An elastic-net penalized Cox regression model combined these connectivity measures with demographic and clinical variables to produce a risk score for AD conversion. The model significantly predicted conversion, with the most predictive regions including heteromodal and visual cortices, caudate, and hippocampus. Risk scores correlated with amyloid and tau PET biomarkers, glucose metabolism, and cognitive/clinical severity, and the model was validated on an independent OASIS dataset with a C-index of 0.716.

More recently, a benchmarking study [27] evaluated several survival models, including a static Cox model, on the ADNI dataset for predicting time to dementia in CN/MCI participants. Notably, the Static Cox baseline model achieved strong performance, with a C-index of 0.901 at the 2-year landmark, 0.885 at 3 years, and 0.856 at 4 years.

To facilitate comparison across studies, a summary is provided below in Table 10.

Overall, these studies suggest that predictive models in AD and MCI cohorts generally achieve C-index values between 0.70 and 0.85, with higher values (>0.85) observed in models leveraging multimodal data, longitudinal features, and advanced ML methods. These findings provide a useful benchmark for assessing the performance of new predictive models in the Alzheimer’s disease context.

Specifically, this work focused on the identification of features capable of distinguishing progressors from non-progressors, highlighting variables that are most informative for predicting disease progression. The results show that strong discriminative performance can be achieved using a minimal set of features, confirming that even a small number of well-selected variables is sufficient to capture key patterns underlying conversion risk. These findings support the use of compact and interpretable models that balance risk stratification performance and model complexity.

## 6. Conclusions

In this study, we developed a robust, data-driven survival analysis pipeline to investigate the progression from MCI and CN to AD, integrating cognitive, radiomic, clinical, and biospecimen features. By leveraging XGBoost for survival data, we were able to handle missing values natively without imputation, ensuring a more reliable modeling of real-world datasets. Our approach incorporated rigorous feature selection and hyperparameter optimization, while emphasizing stability across cross-validation folds and balanced event representation, resulting in a model that is both accurate and interpretable. The study confirms that strong predictive performance can be retained with a small and stable feature set, highlighting the role of model parsimony in prognostic modeling. Cognitive and imaging variables such as AVTOT4, CDR-SB, FAQ, LDELTOTAL, the right middle temporal gyrus thickness and PET_HCI emerged as robust predictors of disease progression, in agreement with previous findings reported in the literature. In particular, the prominence of cognitive measures confirms their well-established role in prognostic modeling of AD. Alongside these cognitive covariates, the lipid metabolite ADMC_HEX2CER.D18.1.20.0. was retained in the selected feature set supporting the complementary contribution of biospecimen data. The selection of this lipid feature is supported by previous studies showing dysregulation in AD, reinforcing the biological plausibility of our findings.

Overall, this work provides a rigorous confirmation of feature stability within a strictly baseline-only survival analysis framework and demonstrates that compact, interpretable models can retain high discriminative performance. By emphasizing transparent feature selection and explicit reporting of the variables used, the proposed framework complements existing literature and may support future translational studies aimed at clinically interpretable risk stratification in AD, pending external validation on independent datasets.

## Figures and Tables

**Table 1:** Event definition based on the transition.

Transition	Event
CN-MCI-AD	1
MCI-AD	1
CN-AD	1
CN-CN	0
CN-MCI	0
MCI-MCI	0

**Table 2:** Information on the dataset and split between train and test.

Parameter	Value
Number of patients in train set	1697
Number of patients in test set	189
Number of events in train set	391
Number of events in test set	43
Percentage of events on the train set (%)	23.04
Percentage of events on the test set (%)	22.75

**Table 3:** Information on the train and validation set in 5 folds.

Fold	Number of Patients in Train Set	Number of Patients in Val Set	Number of Events in Train Set	Number of Events in Val Set	Percentage of Events on the Train Set (%)	Percentage of Events on the Val Set (%)
1	1357	340	313	78	23.07	22.94
2	1357	340	312	79	22.99	23.24
3	1358	339	313	78	23.05	23.01
4	1358	339	313	78	23.05	23.01
5	1358	339	313	78	23.05	23.01

**Table 4:** Hyperparameter exploration and best parameters.

Hyperparameter	Range Explored	Description	Best Parameter
n_estimators	500–2000 (step 250)	Number of boosting iterations	1250
max_depth	2–6	Maximum tree depth	3
learning_rate	0.01–0.1 (log-scale)	Step size shrinkage	0.01
subsample	0.6–1.0	Fraction of samples per tree	0.87
colsample_bytree	0.6–1.0	Fraction of features per tree	0.95
reg_lambda	1–5	L2 regularization	2.12
reg_alpha	0.1–1.0	L1 regularization	0.59

**Table 5:** C-index results on the validation set for each fold using all the available features and on the test set.

Fold	C-Index
1	0.8877
2	0.8794
3	0.8834
4	0.8884
5	0.8822
Mean C-index = 0.8842, STD C-index = 0.0034	
C-index on test set = 0.9059	

**Table 6:** C-index results on the validation set for each fold using the 500 most significant features.

Fold	C-Index
1	0.8991
2	0.8757
3	0.8800
4	0.8934
5	0.8857
Mean C-index = 0.8868, STD C-index = 0.0085	

**Table 7:** C-index results on the validation set for each fold using the 27 features obtained as the intersection across folds and on the test set.

Fold	C-Index
1	0.9024
2	0.8888
3	0.8930
4	0.9032
5	0.9004
Mean C-index = 0.8976, STD C-index = 0.0057	
C-index on test set = 0.9086	

**Table 8:** C-index for each fold considering the 15 common features.

Fold	C-Index
1	0.8952
2	0.8677
3	0.8887
4	0.8845
5	0.8779
Mean C-index = 0.8828, STD C-index = 0.0094	
C-index on Test set = 0.9059	

**Table 9:** C-index for each fold considering the 9 common features.

Fold	C-Index
1	0.7821
2	0.7473
3	0.7495
4	0.7797
5	0.7843
Mean C-index = 0.7686, STD C-index = 0.0166	
C-index on Test set = 0.6767	

**Table 10:** Overview of survival analysis models applied to AD progression prediction across ADNI and related datasets. The table highlights cohort characteristics, feature modalities, modeling approaches, and reported C-index values. Where “−” indicates that the information is not specified.

Study	Test Dataset	Training Dataset	Training Sample Size	Number of Features	Feature Selection Method	Imputation Method	Model	Task	C-Index
Spooner et al. [41]	ADNI-1	ADNI-1	640	207	−	MICE	Cox PH + ElasticNet	CN/MCI to AD	0.93
Kim et al. [42]	OASIS3	ADNI-GO/2/3	115	−	−	−	Cox PH + ElasticNet	CN/MCI to AD	0.716
Shigemizu et al. [5]	NCGG Biobank	NCGG Biobank	98	27	−	IMPUTE2, only for genotype	Cox PH	MCI to AD	0.702
Nakagawa et al. [25]	ADNI, AIBL, JADNI, Shimane	ADNI, AIBL, JADNI, Shimane	1713	246	−	−	Deep Learning Model	CN/MCI to AD	0.835
Jahani et al. [28]	ADNIMERGE	ADNIMERGE	631	14	Cox LASSO	missForest	RSF	MCI to AD	0.878
Abuhantash et al. [29]	ADNI	ADNI	494	43	−	Empirical Sampling	Fast Random Forest	CN to MCI	0.84
Sarica et al. [26]	ADNI-1	ADNI-1	309	43	−	missForest	RSF	MCI to AD	0.89
Ghosh et al. [30]	ADNI	ADNI	577	51	Cox ElasticNet	MICE	Bayesian Model Averaging	CN to MCI/AD	0.608
Signorelli et al. [27]	ADNI	ADNI	1479	−	−	−	Cox PH	CN/MCI to AD	0.901
**Our work**	**ADNI**	**ADNI**	**1697**	**13**	**XGBoost**	**None**	**XGBoost**	**CN/MCI to AD**	**0.92**

## Data Availability

Data used in this work were downloaded from the public dataset available at https://adni.loni.usc.edu/ (accessed on 27 February 2025).
